# A Novel Biodegradable Composite Polymer Material Based on PLGA and Silver Oxide Nanoparticles with Unique Physicochemical Properties and Biocompatibility with Mammalian Cells

**DOI:** 10.3390/ma14226915

**Published:** 2021-11-16

**Authors:** Veronika V. Smirnova, Denis N. Chausov, Dmitriy A. Serov, Valery A. Kozlov, Petr I. Ivashkin, Roman Y. Pishchalnikov, Oleg V. Uvarov, Maria V. Vedunova, Anastasia A. Semenova, Andrey B. Lisitsyn, Alexander V. Simakin

**Affiliations:** 1 Prokhorov General Physics Institute of the Russian Academy of Sciences, Vavilova Str. 38, 119991 Moscow, Russia; veronausckova@mail.ru (V.V.S.); d.chausov@yandex.ru (D.N.C.); dmitriy_serov_91@mail.ru (D.A.S.); v.kozlov@hotmail.com (V.A.K.); ivashkin@kapella.gpi.ru (P.I.I.); rpishchal@kapella.gpi.ru (R.Y.P.); uvarov@kapella.gpi.ru (O.V.U.); mvedunova@yandex.ru (M.V.V.); 2 Department of Fundamental Science, Bauman Moscow State Technical University, 2-nd Baumanskaya Str. 5, 105005 Moscow, Russia; 3 Institute of Biology and Biomedicine, Lobachevsky State, University of Nizhni Novgorod, 23 Gagarin Ave., 603950 Nizhny Novgorod, Russia; 4 V. M. Gorbatov Federal Research Center for Food Systems of the Russian Academy of Sciences, 109316 Moscow, Russia; a.semenova@fncps.ru (A.A.S.); info@fncps.ru (A.B.L.)

**Keywords:** nanocomposite polymer material, PLGA, silver oxide nanoparticles, reactive oxygen species, bacteriostatic properties

## Abstract

A method for obtaining a stable colloidal solution of silver oxide nanoparticles has been developed using laser ablation. The method allows one to obtain nanoparticles with a monomodal size distribution and a concentration of more than 10^8^ nanoparticles per mL. On the basis of the obtained nanoparticles and the PLGA polymer, a nanocomposite material was manufactured. The manufacturing technology allows one to obtain a nanocomposite material without significant defects. Nanoparticles are not evenly distributed in the material and form domains in the composite. Reactive oxygen species (hydrogen peroxide and hydroxyl radical) are intensively generated on the surfaces of the nanocomposite. Additionally, on the surface of the composite material, an intensive formation of protein long-lived active forms is observed. The ELISA method was used to demonstrate the generation of 8-oxoguanine in DNA on the developed nanocomposite material. It was found that the multiplication of microorganisms on the developed nanocomposite material is significantly decreased. At the same time, the nanocomposite does not inhibit proliferation of mammalian cells. The developed nanocomposite material can be used as an affordable and non-toxic nanomaterial to create bacteriostatic coatings that are safe for humans.

## 1. Introduction

Bacterial antibiotic resistance is an urgent problem of world health care. In the world, the death of patients due to antibiotic-resistant bacteremia exceeds total death from diabetes mellitus and cancer [1]. One of the ways to overcome antibiotic resistance is to use metal oxides nanoparticles (NPs) [2], in particular, silver oxide NPs [3]. The presumptive mechanisms of the antibacterial action of NPs of metal oxides are ROS generation, violation of the integrity of the bacterial cell wall, inhibition of ATPase activity, genotoxic and photocatalytic actions, violation of bacterial DNA replication processes, and suppression of the expression of numerous genes [4,5,6,7,8,9,10,11]. The silver oxide nanoparticles advantages are high antibacterial efficiency, good biocompatibility with mammalian cells, and low synthesis cost [12,13,14].

To prolong the antibacterial action, nanoparticles are often coated with polymers: polyethylene sulfone, chitosan, polyethylene terephthalate, etc. [15,16,17,18]. In addition, polymer films supplemented by metal oxide NPs and the surfaces of such films acquire antibacterial properties [19,20,21,22,23]. At present, such nanocomposites are already used to create cover, textile, and even building materials with antibacterial properties [23,24]. Nano- and micromaterials contained of poly (lactic-co-glycolic acid) (PLGA) are promising candidates for many biomedical applications, in particular, oncology therapy, targeted drug delivery, and vaccine efficacy improvement [25,26,27]. PLGA is often used to make biodegradable stent coatings, as well as parts of the stents themselves and other materials used for implantation [28,29,30,31,32,33,34,35,36].

Obviously, the range of applications for PLGA-based materials can be significantly expanded by acquiring new exclusive properties. The idea of creating a material based on PLGA and silver is not new. Previously, several materials with silver salts [33], silver nanoparticles [34], and even silver fibers [35] were prepared. Nanocomposites based on PLGA and Ag NPs demonstrate good bio-compatibility to mammalian cells on the one hand, and antibacterial action, on the other [37,38]. In addition, PLGA/Ag NPs nanocomposites are biodegradable, so they can be used in surgery [39]. The synthesis of a polymer in the presence of silver NPs allows one to control the physicochemical nature of the resulting composire’s properties. It expands the possible range of their application [40,41].

Silver oxide nanoparticles have one important advantage over metallic silver nanoparticles [37]. Nanoparticles of their silver oxide dissolve better in aqueous solutions and, as a consequence, lead to a more rapid increase in the concentration of silver ions in aqueous solutions. Currently, an active search is under way for ways to improve the antibacterial properties of PLGA/Ag NPs nanocomposites, including a modification of the method for synthesizing nanoparticles [42]. It should be noted that most materials based on metallic silver nanoparticles did not have the best mechanical and physicochemical properties, and the nanoparticles themselves were randomly distributed in the material. In the present study, using laser ablation, we synthesized silver oxide nanoparticles and fabricated a nanocomposite with a gradient concentration of nanoparticles in the bulk.

Laser ablation is a method of synthesizing NPs of metals and their oxides by laser irradiation of a target metal contained in a solvent. This method makes it possible to fairly finely control the size of the resulting NPs due to the synthesis conditions (radiation parameters and the solvent used) [43,44,45,46]. Additional advantages of laser ablation are its high speed and low cost of synthesis [47]. The unusual physicochemical properties of the nanocomposite have been investigated, its bacteriostatic activity has been determined, and its biocompatibility has been studied.

## 2. Materials and Methods

### 2.1. Silver Oxide Nanoparticles Preparation and Characterization

**Figure 1:**
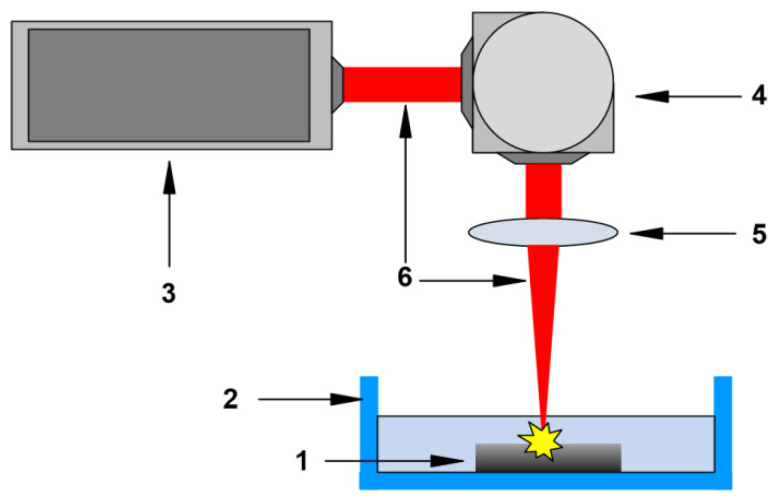
Schematic diagram of a laser ablation setup. 1—Colloid of Ag_2_O Nps in water; 2—Cuvette in a water-cooled cell; 3—Nd: YAG laser; 4—Galvano-mirror system; 5—F-theta objective; and 6—laser beam.

Silver oxide nanoparticles were synthesized by laser ablation in liquid with pulsed ytterbium-doped fiber laser (Pokkels, Moscow, Russia). We used commercially available Ag ≥ 99.9% silver (SigmaAldrich, Saint Louis, MO, USA) as a target metal. The target metal was placed in a container with 10 mL of deionized Milli-Q water (with a resistivity of at least 18 MΩ/cm) so that the upper surface of a piece of metal was immersed in water by ~1 mm. The target metal was irradiated with a laser beam directed from top to bottom. The tank was continuously cooled using a water-cooling system. The laser beam was focused using an F-theta objective (F = 10 cm). The calculated diameter of the laser beam at the waist area was ~50 μm. To ensure uniform irradiation of the target metal surface, the position of the laser beam was constantly changed using a galvo-mirror system with a scanning speed of 500 mm/s, and the area of the irradiation zone was ~1 cm^2^. The time and parameters of the irradiation were selected in preliminary experiments [48]. Parameters of laser pulses were λ = 1064 nm, τ = 4–200 ns, energy 2 mJ, average power ≤ 20 W, and repetition frequency 20 kHz. The irradiation time was 10 min. The schematic diagram of the laser ablation setup is shown in Figure 1.

To obtain nanoparticles uniform in size, the resulting colloid of nanoparticles was centrifuged in an analytical centrifuge. The speed and time of centrifugation were selected in such a way as to precipitate NPs with a size of more than 60 nm for their subsequent removal. The distribution of Ag NPs on size and zeta potential was evaluated by dynamic light scattering method with Zetasizer Ultra Red Label (Malvern Panalytical, Malvern, UK). The measuring features were described earlier [49]. The size, shape, surface structure, and element composition of nanoparticles were studied with Libra 200 FE HR transmission electron microscope (Carl Zeiss, Jena, Germany). Atomic force microscopy was performed using an SII Nanopics 2100 atomic force scanning microscope (KLA-Tencor, Milpitas, CA, USA), in the dynamic force microscopy (DFM) mode. The NPs aqueous colloids spectra were obtained with Cintra 4040 spectrophotmeter (GBC Scientific Equipment, Braeside, Australia). The features of the optical spectrum have been described in detail earlier [50].

### 2.2. Composite Fabrication, Production of Plates from Composite Material, and Rheological Properties

The PLGA-based composite with silver oxide NPS was synthesized by the low-temperature technology developed by us earlier [47]. Production of plates from composite material. The PLGA-based material was heated to 40 °C and rolling up to 1000 μm thickness film. Further films were cut into 20 mm × 25 mm (area 10 cm^2^) rectangle piece. Each film piece was placed in 20 mL water. Nanoparticles were added to the material at the stage of polymerization at concentrations of 0.001, 0.01, or 0.1% of the weight of the polymer matrix [51].

### 2.3. Thermal Characteristics Assay

Thermal characteristics assay was carried out by differential scanning calorimetry with DSC 3 Excellence (Mettler Toledo, Columbus, OH, USA) [52]. Thermograms in the heating and cooling modes were constructed to assess thermal characteristics. The temperatures of glass transition (*Tg*) and heat capacity change (Δ*C_p_*) were also evaluated at different dopant conentrations.

### 2.4. Hydrogen Peroxide Concentration Measurement

The measurement of hydrogen peroxide concentration was carried out by highly sensitive modified chemiluminescence with Biotox-7A-USE ultrasensitive chemiluminometer (ANO Engineering Center—Ecology, Moscow, Russia). This method is based on luminol oxidation by horseradish peroxidase in presence of *p*-iodophenol [48]. The measurement and calibration procedure was described earlier for different cases [53,54,55]. The samples were placed in 1 mL of a “counting solution” (1 µM Tris-HCl buffer with pH 8.5, 50 μM *p*-iodophenol, 50 μM luminol, 10 nM horseradish peroxidase). The “counting solution” was prepared immediately before the measurement. The minimum H_2_O_2_ concentration evaluated by modified chemiluminescence method is <1 nM [55].

### 2.5. Hydroxyl-Radicals Concentration Measurement

Concentrations of hydroxyl-radicals were measured by fluorescence of 7-hydroxycoumarin-3-carboxylic acid (7-OH-CCA) [56]. 7-OH-CC is a product of coumarin-3-carboxylic acid (CCA) hydroxylation in presence of OH-radicals. Experimental samples and negative control were heated at 80 ± 0.1 °C for 2 h in mixture of 0.5 mM CCA water solution (pH 3.6) and 0.2 M phosphate buffer (pH 6.8) The 7-OH-CCA fluorescence intensity was measured with spectrofluorimeter JASCO 8300 (JASCO, Tokyo, Japan) at wavelengths 400/450 nm (ex/em). Calibration curve was built with using commercially available 7-OH-KKK [57]. 

### 2.6. Mesurement of Long-Lived Reactive Protein Species Concentrations

Concentrations of long-lived reactive protein species (LRPS) were measured by chemiluminescence of X-irradiated protein solutions [58,59]. In this method, free radicals amount evaluated by light quanta emission during free-radical interactions. The experiments were carried out at room temperature, in the dark, and in plastic polypropylene vials for liquid scintillation counting (Beckman, Brea, CA, USA). We used large volumes (20 mL) in comparison with standard volume (0.1 mL) to improve (in ≤200 times) the sensitivity of method [60]. All samples were irradiated by X-rays and incubated 30 min after X-rays exposure, and chemiluminescence intensities were measured with Biotox-7A-USE chemiluminometer (ANO Engineering Center—Ecology, Moscow, Russia). The non-treated by X-radiation proteins were used as controls. The detailed description of method may be found in [61].

### 2.7. Mesurement of 8-Oxoguanine Concentration

Concentrations of 8-oxoguanine in samples were measured by a non-competitive enzyme-linked immunosorbent assay (ELISA) with anti-8-oxoguanin monoclonal antibodies [62]. Before measuring, samples of DNA (350 μg/mL) were boiled 5 min in water bath for and cooled 3–4 min in ice to induce DNA denaturation. Each sample (42 μL) was added into well of 98-well plate. Wells with samples were dried by incubation 3 h at 80 °C to adsorb DNA on plate bottom surface. Blocking of nonspecific binding was carried out by incubation of samples with 300 μL of 1% skimmed milk (in 0.15 M Tris-HCl buffer, pH 8.7, supplemented by 0.15 M NaCl) overnight and at room temperature. Wells were washed twice (300 μL/well) with 50 mM Tris-HCl buffer (pH 8.7) supplemented by 0.15 M NaCl and 0.1% Triton X-100. Further, 100 μL/well of anti-8-OG antibodies in appropriate dilution was added in each well. Wells were washed twice as described previously after 20 min incubation. Further samples were incubated in 80 μL/well solution of horseradish peroxidase-conjugated with secondary antibodies (1:1000 dulition) in blocking buffer and were washed 3 times as described previously. Subsequently, 100 μL of chromogenic substrate (18.2 mM ABTS and hydrogen peroxide (2.6 mM) in 75 mM citrate buffer, pH 4.2) was added to each well [63]. After 15 min incubation, 50 μL/well of 1.5 mM NaN_3_ was added to 0.1 M citrate buffer (pH 4.3) to stop the reaction [63]. The optical density of samples at 405 nm was evaluated with photometer (Titertek Multiscan, Vantaa, Finland). The method was described in more detail earlier.

### 2.8. Assay of Bacteriostatic Activity

Bacteriostatic activity was evaluated by experiments with Gram-negative *Escherichia coli* bacteria (LenReaktiv, St. Petersburg, Russia). *E. coli* were cultured and subcultured in LB medium in sterile solution by standard protocol [58]. Bacterial cells concentrations in liquid medium were evaluated by spectrophotometry with drop UV5Nano Excellence (Mettler Toledo, Columbus, OH, USA). Equal amount of BL medium with same concentration of *E. coli* was inflicted on previously sterilized with 70% ethanol nanocomposite film (size 10–15 mm and 1000 μm thickness) in sterile hoop and sealed by glass slide. Bacteria were cultures 24 h, at 37 °C, approximately 150 rpm in shaker incubator ES-20 (Biosan). After incubation, concentration of bacteria was measured by spectrophotometry as described above [64].

### 2.9. Assay of Biocompatibility with Mammalial Cells

Studies of biocompatibility were carried out on SH-SY5Y cell line of human neuroblastoma. The SH-SY5Y is a standard cell model. SH-SY5Y cell line is a subclone from the SK-N-SH cells, which were isolated from the four-year-old female patient with neuroblastoma. The differentiation is the process of implementing a genetically determined program for the formation of a specialized cell phenotype, reflecting their ability regarding certain profile functions. SH-SY5Y cells are also interesting in that they can grow not only in monolayers but also form cell aggregates, which also take root on substrates SH-SY5Y usually used in the study of cells proliferation and differentiation in different conditions. For example, SH-SY5Y cells can spontaneously divide into one of two phenotypes, similar to “neuroblast” or “epithelial” phenotypes [65]. The SH-SY5Y cells were cultured by standard protocol in DMEM (Biolot, Moscow, Russia) supplemented with 10% fetal bovine serum (Gibco, USA) and 30 μg/mL gentamicin in a CO_2_ incubator (Binder, Tuttlingen, Germany). SH-SY5Y cell (10^4^ cells/cm^2^) were seeded on material samples (20 mm × 20 mm) in 35 mm Petri dishes (1 sample/dish) in 3 mL cell culture medium and cultured for 3 days in CO_2_ incubator. Cells viability assay were carried out by fluorescent microscopy. Cells were stained for 10 min at 37 °C by 2 μg/mL Hoechst 33342 (Sigma, Saint Louis, MO, USA) to indicate all cells and 2 μg/mL propidium iodide (Sigma, USA) to indicate death cells (Figure 1). Stained cells were washed by phosphate buffer and analyzed with confocal microscope Leica DMI6000 (Leica, Munich, Germany). At least 500 cells were analyzed in each sample [66].

**Figure 2:**
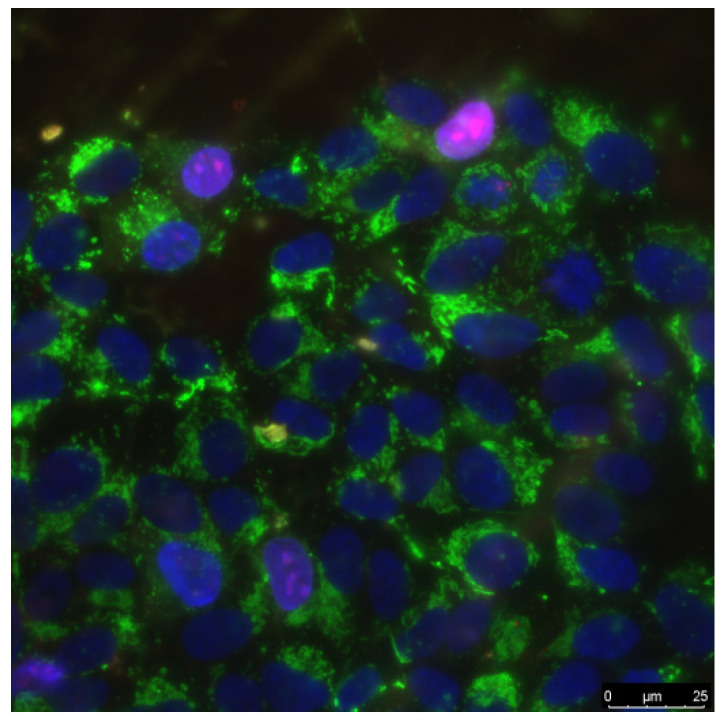
The sample of cell culture micrograph. The mitochondria of the cells are colored green and allow one to determine the size of cells. Normal cell nuclei are colored blue. Nuclei of non-viable cells are stained in purple.

Proliferation assay was carried out on cells in logarithmic growth phase (3 days of growth on nanocomposite). We used a number of cells in a state of mitosis (mitotic index) to estimate cell proliferation. Cells were stained with the Hoechst 33342 fluorescent dye at 15 min and further analyzed by fluorescence microscopy. Depending on distribution of chromatin in nuclei, cells were categorized into mitosis stage: prophase (P), metaphase (M), anaphase (A), and telophase (T). On each sample surface, at least 500 cells were analyzed. The mitotic index (MI) was calculated by the formula MI = (P + M + A + T)/N × 100%, where P, M, A, and T are the numbers of cells at the stage of prophase, metaphase, anaphase, and telophase, respectively, and N is the total number of analyzed cells [67]. A typical stained cell culture specimen is shown in Figure 2.

### 2.10. Statistic

The all data are presented as means ± SE. Data from at least three independent experiments were used in each experimental condition. Significance of differences between samples means was evaluated with two sample two-tailed *t*-tests. GraphPad Prims (GraphPad Software, San Diego, CA, USA), Origin (OriginLab Corporation, Northampton, MA, USA), and SigmaPlot (Systat Software Inc., San Jose, CA, USA) software were used to data statistical processing.

## 3. Results

**Figure 3:**
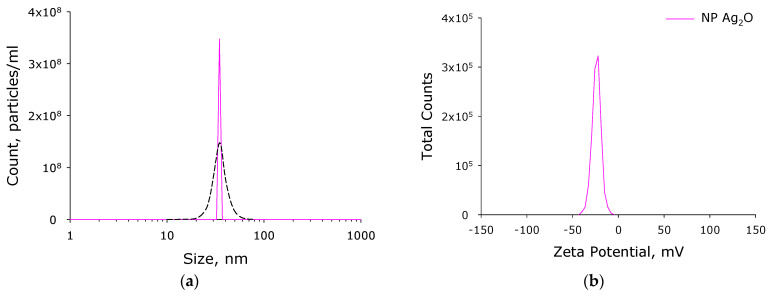

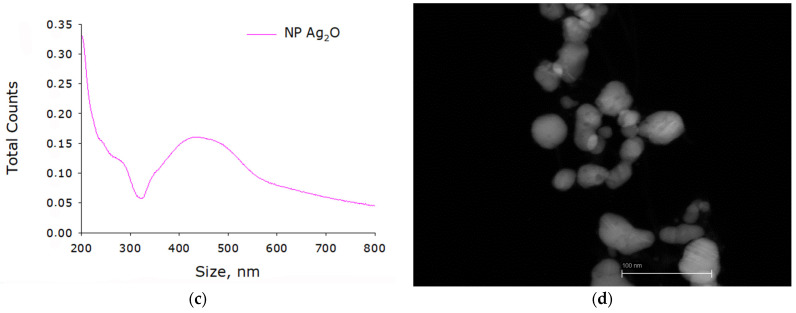
Physicochemical properties of silver oxide NPs. (**a**) Concentration (DLS, solid crimson line) and size distribution (CPS, black dashed line) of silver oxide NPs. (**b**) ζ-potential of Ag_2_O NPs. (**c**) Example of absorption spectrum of colloidal solution of Ag_2_O NPs. (**d**) Example of Ag_2_O NPs TEM image.

Ag_2_O NPs were successfully synthesized by laser ablation in water. The concentration of nanoparticles in the working fluid of the ablation reactor was determined using the DLS Malvern Ultra Red Label (Figure 3a) and was almost 350 million particles per mL. The nanoparticle size distribution is monomodal and rather narrow (Figure 3a). The hydrodynamic diameter of nanoparticles is ~35 nm, and the half-width of the distribution is 25–45 nm. The distribution of nanoparticles by zeta potential values is also monomodal (Figure 3b). The average ζ-potential of nanoparticles is −25 mV (from −8 to −40 mV). The absorption spectrum of an Ag_2_O NPs colloid is demonstrated in Figure 3c. It is shown that the absorption spectrum of nanoparticles corresponds to the absorption spectrum of silver oxide nanoparticles. The size of the nanoparticles was independently verified by TEM (Figure 3d). It is shown that the bulk of the nanoparticles in the photographs have a size from 20 to 40 nm.

**Figure 4:**
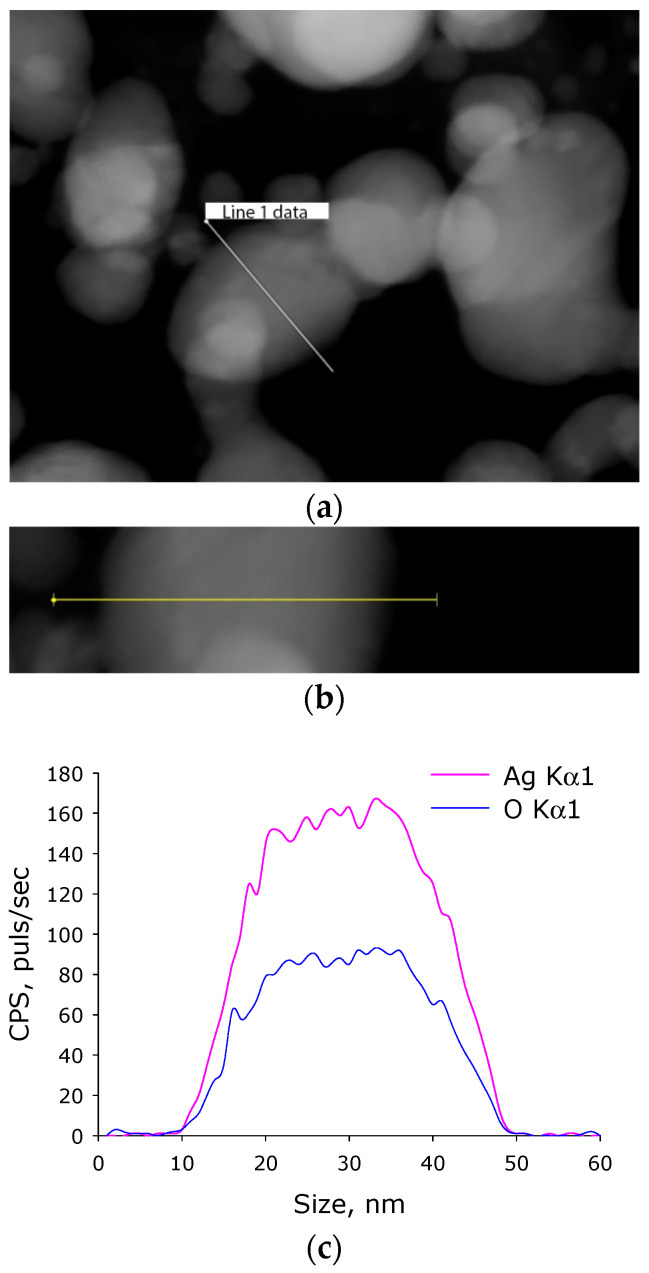
Elemental analysis of of silver oxide NPs. (**a**) TEM image of group of silver oxide NPs; analysis section is indicated by line 1. (**b**) Enlarged measurement site. (**c**) Nanoparticle profile by Ag Kα1 and O Kα1.

Silver is known to form several different oxides. The elemental composition was evaluated by energy dispersive X-ray spectrometry (Figure 4). It was found that the nanoparticles obtained by us consist of two chemical elements: silver and oxygen. The ratio of atoms Ag/O = 1.94; thus, it can be assumed that the nanoparticles are predominantly composed of Ag_2_O.

**Figure 5:**
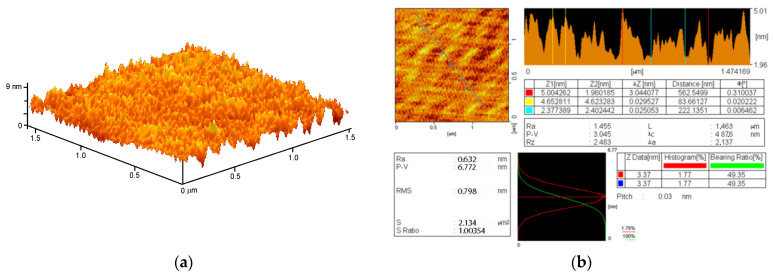
Reconstruction of the surface of a polymer and composites based on it, performed using an atomic force microscope. (**a**) 3D reconstruction; (**b**) surface analysis results.

The resulting NPs were transferred into PLGA polymer using a previously developed low-temperature technology [47]. The composite PLGA/Ag_2_O NPs had visually smooth surfaces. The surface relief was studied using atomic force microscopy (Figure 5a). It is shown that the composite material has no significant flaws, cavities, holes, cracks, or breaks. Both the analytical slow scan and the high-speed scan did not reveal any defects. The roughness of the test samples was ≤0.633 nm (Figure 5b).

Atomic force microscopy provides information on the surface topology but does not answer the question of the relative position of both polymer and nanoparticles in the composite material. It is known that PLGA and silver oxide differ significantly in their optical properties from each other. In this regard, we applied modulation interference microscopy, which allows us to isolate patterns in materials that differ in refractive index and other optical properties. The refractive index was determined at the wavelength of the laser microscope.

**Figure 6:**
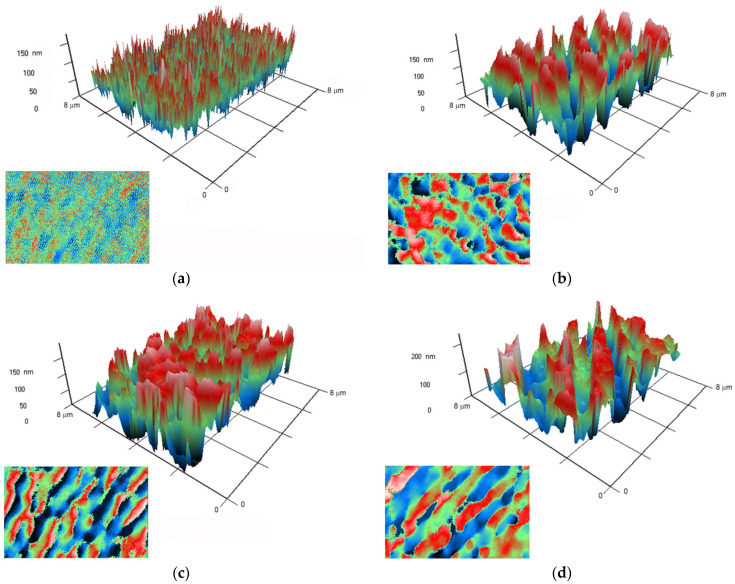
Images obtained on polymer without nanoparticles (**a**), and polymers with Ag_2_O nanoparticles at concentrations of 0.001% (**b**), 0.01% (**c**), and 0.1% (**d**), using a ja. A 3D reconstruction of the surface profile of a polymer and composites based on it is presented. The X and Y axes show the actual size of the investigated surface in micrometers. The Z-axis shows the surface relief as a phase change expressed in nm. The surface elevation map is in left lower part in each picture.

The refractive index of the unmodified PLGA is 1.47 at 405 nm, and the refractive index of the silver oxide is 1.19 at 405 nm. Thus, the refractive index of PLGA and silver oxide nanoparticles differs by almost 0.3 units. It has been shown that PLGA without nanoparticles does not have any pronounced surface structure (Figure 6a). When silver oxide nanoparticles are added to the polymer at a concentration of 0.001%, the composite surface breaks down into domains that differ significantly in the change in the phase of the laser radiation (Figure 6b). It should be noted that domains are rather large, on average 0.5 μm × 0.5 μm, and domains of much larger sizes are encountered. The domains begin to merge with each other, forming elongated structures several micrometers long, during increasing of dopant concentration in the polymer to 0.01 or 0.1% (Figure 6c,d). The data obtained make it possible to assert that the nanoparticles in the polymer are not evenly distributed. It can be assumed that domains with a large phase incursion are the centers of concentration of nanoparticles in the polymer.

**Figure 7:**
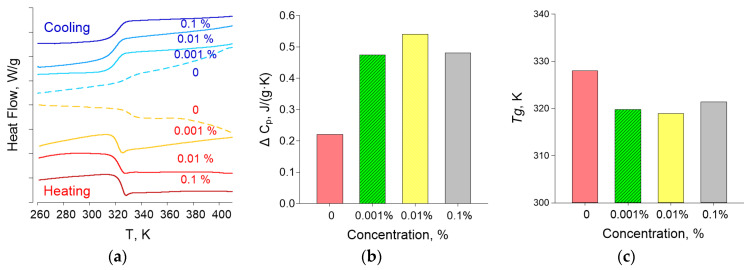
Thermal analysis of nanocomposites using differential scanning calorimatry: (**a**) thermograms obtained in heating and cooling modes; (**b**) glass transition temperatures of the samples; and (**c**) change in heat capacity of the test samples.

Thermograms of the composite material obtained in the heating and cooling mode are shown in Figure 7a. The glass transition of the material is clearly visible 320–325 K, which is observed for all samples. Based on the results of differential scanning calorimetry, the glass transition temperatures *Tg* and the change in heat capacity Δ*C_P_* of the samples under study were determined, the concentration dependences of which are shown in Figure 7b,c, respectively. The glass transition temperature is in the range 319–321 K and corresponds to the literature data for pure PLGA [52]. The Δ*C_P_* values vary within 7% of 0.5 J/(g × K), which is comparable to the measurement error.

**Figure 8:**
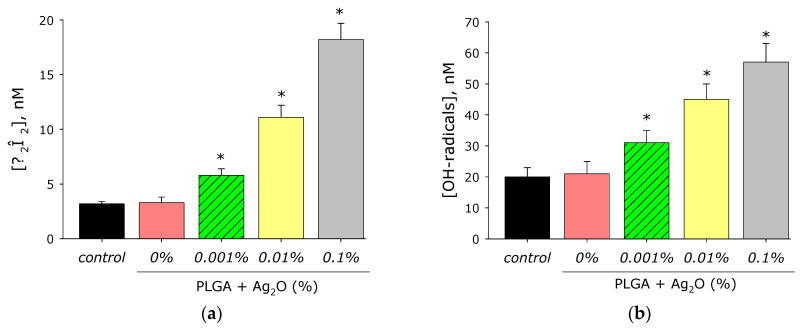
Effect of composite material containing PLGA and Ag_2_O nanoparticles on the generation of reactive oxygen species: (**a**) H_2_O_2_ generation (2 h, 40 °C); (**b**) generation of OH-radicals (2 h, 80 °C); *—*p* < 0.05 versus control. Data are presented as mean ± ME.

It is known that metals of variable valence in aqueous solutions and biological fluids often lead to the generation of ROS. The effect of PLGA/Ag_2_O NPs on ROS generation such as H_2_O_2_ (the longest-lived ROS) (Figure 8a) and OH-radicals (the most active ROS) (Figure 8b) was investigated. PLGA-based did not influence the generation of H_2_O_2_ in an aqueous solution. Composite PLGA/Ag_2_O NPs at all concentrations of nanoparticles increase the rate of H_2_O_2_ generation.

Addition of Ag_2_O NPs at concentration of 0.001 in composite increases rate of H_2_O_2_ generation in comparison with the control by almost two times. The rate of H_2_O_2_ generation increases more than three times at Ag_2_O NPs concentration of 0.01% and almost five times at concentration 0.1%. The effect of the composite material on the generation of hydroxyl radicals has been investigated. PLGA polymer did not influence the generation of OH-radicals. In this case, composite PLGA/Ag_2_O NPs significantly increase OH-radicals generation.

**Figure 9:**
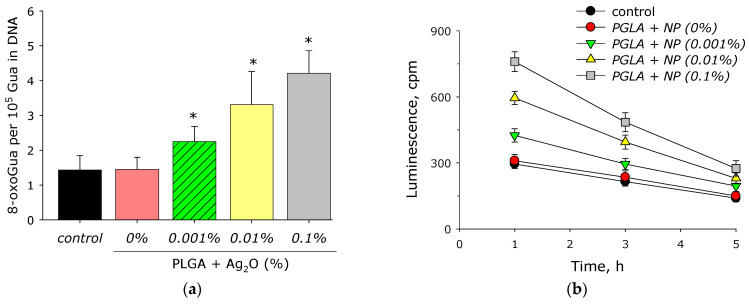
Effect of composite PLGA/Ag_2_O NPs on the biomacromolecules damage formation: (**a**) generation of 8-oxo-G in DNA in vitro (2 h, 45 °C); (**b**) formation and dynamics of decomposition of long-lived reactive protein species (2 h, 40 °C); *—*p* < 0.05 versus control. Data are presented as mean ± SE.

Elevated generation of ROS often leads to biomacromolecule modification or destruction. The effect of composite PLGA/Ag_2_O NPs on the formation of 8-oxoguanine in DNA was evaluated (Figure 9a). PLGA did not change the rate of 8-oxoguanine generation. Addition of Ag_2_O NPs to the polymer significantly increased the rate of 8-oxoguanine generation. At a concentration Ag_2_O NPs of 0.001%, the rate of 8-oxoguanine generation increases by 60% compared to the control, at a concentration of 0.01% by 130%, and at a concentration of 0.1%, by almost three times.

The effect of composite PLGA/Ag_2_O NPs on the formation of LRPS was studied (Figure 9b). It was shown that PLGA containing no silver oxide nanoparticles did not influence on LRPS generation or LRPS decay rate. It was found that when Ag_2_O NPs appear in the polymer, the rate of LRPS generation increases significantly. The increasing in the rate relative to the control by about 40% is observed at a concentration of silver oxide nanoparticles of 0.001%. With an increase in the concentration of Ag_2_O NPs to 0.01%, the rate of LRPS generation increases by almost two times, at a concentration of 0.1%—more than 2.5 times. At the same time, silver oxide nanoparticles have almost no effect on the average half-life of active forms of proteins. In all groups, the half-life is about 4–5 h.

**Figure 10:**
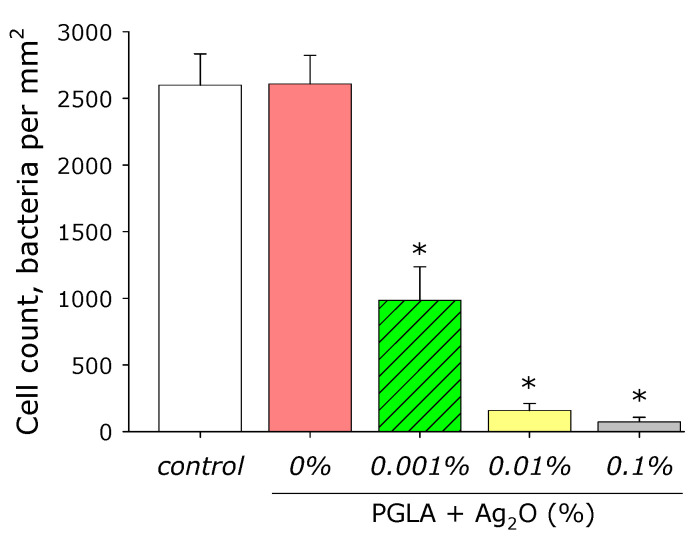
Influence of composite PLGA/Ag_2_O NPs on the *E. coli* growth (bacteriostatic effect). *—*p* < 0.05 versus control. Data are presented as mean ± SE.

The effect of composite PLGA/Ag_2_O NPs on *E. coli* growth was studied (Figure 10). It was shown that PLGA containing no silver oxide nanoparticles did not influence on *E. coli* bacteria growth. The addition of Ag_2_O NPs in the polymer at a concentration of 0.001% decreased the density of bacterial cultures grown by more than half compared to the control, at a Ag_2_O NPs concentration of 0.01% (by almost 95%), and at a Ag_2_O NPs concentration of 0.1% (by more than by 97%).

**Figure 11:**
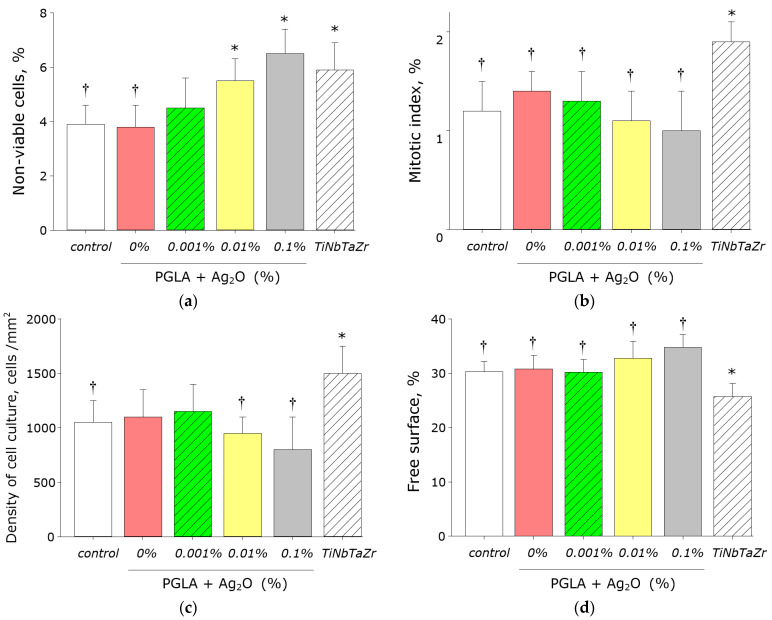
Effect of composite PLGA/Ag_2_O NPs on the main characteristics of growth and development of cell culture: (**a**) Influence of composite material on the viability of SH-SY5Y cell culture; (**b**) Influence of composite material on the SH-SY5Y cells mitotic index; (**c**) Influence of composite material on the cells SH-SY5Y density; (**d**) Influence of composite material on the colonisation rate of free surface by cells. *—*p* < 0.05 versus control. †—*p* < 0.05 versus NiNbTaZr group. Data are presented as mean ± SE.

The effect of composite PLGA/Ag_2_O NPs on the viability of eukaryotic cells was investigated (Figure 11a). The almost of non-viable cells on control substrates (culture plastic) was no more 4%. Approximately the equal almost of death cells was observed on PLGA without silver oxide nanoparticles or 0.001% silver oxide nanoparticles. At the same time, when using the TiNbTaZr medical alloy as a substrate, an almost one and a half greater number of nonviable cells was determined (almost 6%). Number of nonviable cells were about 5.5% of the cells in case of PLGA and silver oxide nanoparticles (0.01%) and 6.5% of the cells in case of PLGA and silver oxide nanoparticles (0.1%).

The influence of the composite material on the mitotic index (percentage of cells in a dividing state) was investigated (Figure 11b). The number of proliferated cells was determined by fluorescence microscopy. The mitotic index for cells cultured on the culture plastic is 1.2%. In the case of medical alloy TiNbTaZr, the mitotic index is almost 2%. When cells were grown on composite PLGA/Ag_2_O NPs, the mitotic index was 1.0–1.4%, which did not statistically differ from the control values.

Density of the cell culture growth on plastic is on average 1000 cells/mm^2^ of surface (Figure 11c). The cell culture density reaches almost 1500 cells/mm^2^ in case of grown on TiNbTaZr medical alloy. When cells were grown on composite PLGA/Ag_2_O NPs, the density of the cell culture was 800–1100 cells/mm^2^, which did not statistically differ from the control values.

The surfaces of composite PLGA/Ag_2_O NPs have been shown to be suitable for cell life, growth, and proliferation (Figure 11d). Moreover, the degree of suitability is comparable to that of control. At the same time, the degree of suitability of the composites is somewhat lower than that of the TiNbTaZr medical alloy. With the duration of the experiment for 72 h of growth, a completely confluent monolayer of cells is not formed on all materials. Only individual elements of the monolayer formed. Maximum confluence of cells on all studied material was about 70–75% of the surface area.

## 4. Discussion

The average size of the synthesized NPs was 25–50 nm (Figure 3a,d). In our work, the average NP size is somewhat smaller than in most other works [16,17,18,22,68]. This tendency can be explained by the differences in the method of synthesizing silver oxide NPs: in these studies, the coprecipitation method and polymer matrices were used that were different from PLGA. The average value of the ζ-potential of the NPs synthesized by us is –30 mV (Figure 3b), which corresponds to stable NPs [69]. The absorption spectra with a peak at 450 nm correspond to silver oxide NPs [20,22]. According to TEM data with elemental analysis, we obtained an Ag:O ratio of ~2:1 (Figure 4), which indicates that the nanoparticles we obtained mainly consist of silver (I) Ag_2_O oxide. The modulation interference microscopy method, in contrast to atomic force microscopy (Figure 5), allows one to assess the uneven distribution of nano- and micro-sized particles [70]. Using modulation interference microscopy, we found that NPs were unevenly distributed over the polymer volume (Figure 6).

The addition of nanoparticles to PLGA alters the behavior of the polymer during heating and cooling, increases the temperature of glass transition (*Tg*), and decreases the composite heat capacity compared to the polymer without a dopant (Figure 7). An increase in the temperature of glass transition, and heat-capacity decrease, may indicate a decrease in the internal viscosity of the polymer [52]; thus, the addition of Ag_2_O NPs modifies the physical properties of the nanocomposite. The largest drop in the glass transition temperature and an increase in heat capacity were observed at a dopant concentration of 0.01%. The results show that the *Tg* can be adjusted by changing the volume fraction of the nanoparticles. With an increase in the volume fraction of NPs, a decrease in *Tg* is observed first and then an increase in *Tg*. The nonlinear dependence of *Tg* and thermal conductivity of the nanocomposite on the dopant concentration was found by us in previous studies and is described in the literature [71,72]. The nonlinear character of the dependence of the *Tg* of the nanocomposite on the dopant concentration was also found in mathematical modeling [73]. According to the literature, the main mechanism of the complex change in *Tg* with increasing NPs concentration is competition between the attraction of nanoparticles to polymer chains and rapid diffusion of nanoparticles at the initial stage of polymer composite synthesis [73]. The aggregation of NPs with an increase in their concentration in the polymer matrix can also be one of the mechanisms for changing the properties of the polymer matrix. Aggregation of Ag_2_O NPs is noted during the synthesis by the coprecipitation method [74]. In addition, we detected the formation of Ag_2_O NPs clusters in the nanocomposite by the MIM method, which indicates the role of NP aggregation in the polymer in the regulation of the physical properties of the nanocomposite (Figure 6).

ROS generation is a general mechanism of nanomaterial toxicity. Excess ROS generation lead oxidative cell stress and disturbance of redox functions and metabolism [75,76]. However, ROS may play physiological role in cell signal transduction and regulation of cell responses to mitogens [77]. PLGA-based material does not influence H_2_O_2_ and OH-radical generation (Figure 8). This fact differs PLGA from some other polymer material, which can increase ROS generation rate [78,79]. Therefore, PLGA is more suitable polymer matrix to development of biocompatible nanocomposites. Development of oxidative stress includes oxidative protein modification, protein radical generation [80], and lipid peroxidation [81].

The presented Ag_2_O NPs in composite increase the rate of H_2_O_2_ generation by almost 2 and 5 times at a percentage of nanoparticles 0.001–0.1% (Figure 8a). This behavior of silver oxide nanoparticles in redox reactions leading to ROS generation is in agreement with the works [82,83]. PLGA does not influence OH-radical generation (Figure 8b). Addition of Ag_2_O NPs to PLGA increases rate of OH-radical generation by 1.5–3 times, which differs from literature data [84,85]. 8-oxoguanine is an important marker of DNA oxidative damage; therefore, 8-oxoguanine generation in DNA is of great interest in medicine [86]. 8-oxoguanine can lead to the formation of mismatched nucleotides with adenine and thus lead to GC-TA transversion, which increases risk of mutations. At least four reparation pathways are realized in mammals for deleting 8-oxoguanine from DNA and for preventing its introduction into DNA. The duplicating of protection mechanisms indicates that 8-oxoguanine is an extremely serious danger for cells and that it must be quickly eliminated [87]. (Figure 9a) PLGA did not influence 8-oxoguanine generation in DNA, which differed from literature data [88,89]. The addition of silver oxide nanoparticles in PLGA significantly increases the rate of 8-oxoguanine generation. The rate of 8-oxoguanine generation increases by 1.4, 2.2, and 3 times at a concentration of Ag_2_O NPs of 0.01, 0.01, and 0.1%, respectively. LRPS are not only an important marker of oxidative stress but can also generate secondary radicals [90,91]. PLGA without nanoparticles did not increase the generation of LRPS (Figure 9b). The addition of Ag_2_O NPs increased the generation of LRPS by 25 (for 0.001% NPs), 98 (for 0.01% NPs), and 152% (for 0.01% NPs), compared to control. The generation of hydrogen peroxide, hydroxyl radical, 8-oxoguanine, and LRPS may be the mechanisms of the antimicrobial effects of the PLGA/Ag_2_O NPs nanocomposite [92].

In some cases, biomaterial may be substrate to microorganisms and infection source itself [89,92]. For example, bacteria from air and skin can contaminate medical devices and induce hospital-acquired infections [93].

Microorganisms can grow, forming biofilms on surface of some biomaterials [94]. We observed that (Figure 10) PLGA, without silver oxide nanoparticles, did not affect *E. coli* growth. Bacterial growth decreased on polymer with silver oxide nanoparticles. Bacterial cultures density over the composite decreased by 65, 94, and 98% at a silver oxide nanoparticles concentration of 0.001, 0.01, and 0.1%, respectively. In our study, we proposed that 0.001% of silver oxide nanoparticles is similar to minimum inhibitory concentration (MIC) 10 µg/mL. MIC, in case of silver oxide nanoparticles coated other by polyethersulfone, cellulose, and hydrogel, is above 2–8000 µg/mL (Table 1) [17,18,19,95,96].

Moreover, the nanocomposite is suitable for anchoring and spreading mammalian cells (Figure 11). The nanocomposite has comparable biocompatibility (number of living cells, values of mitotic index, and density of cell culture and free surface) with the medical alloy TiNbTaZr or special plastic utensils for growing cells. The PLGA/Ag_2_O NPs nanocomposite obtained by us is a promising candidate for the development of materials with high antibacterial activity and good biocompatibility with human cells. Such materials can find application in surgery, in particular, in prosthetics.

## Figures and Tables

**Table 1:** Antimicrobial properties of polymers/Ag_2_O nanocomposites.

Polymer Matrix	D, nm	Microorganism Strains	MIC	Ref.
Chitosan	50–500	*B. subtilis* ATCC 6538, *E. coli* MTCC 1303, *P. aeruginosa* ATCC 6633, *S. aureus* MTCC 2453	5 mg/mL	[16]
Polyethersulfone (PES)/cellulose acetate (CA)	20–100	*E. coli*	8 mg/mL	[17]
Polyethylene terephthalate (PET)	50–500	*E. coli*	-	[18]
Natural hydrogel from *Abroma augusta*	20–40	*Bacillus cereus* MTCC 430, *E. coli* MTCC 443, *Klebsiella pneumoniae* MTCC 7162, *P. aeruginosa* MTCC 741, *S. aureus* MTCC 96	12.5 µg/mL	[19]
Chitosan	50–100	*E. coli*, *S. aureus*	2 µg/mL	[23]
Polyvinyl alcohol (PVA) or starch (aspirin conjugated)	-	*Alternaria solani*, *A. niger*, *Citrobacter freundii*, *Curvularia lunata*, *Enterobacter aerogenes*, *E. coli*, *Helmentiasporium maydis*, *Paecilomyces lilacinusby*, *P. vulgaris*, *Rhizopus nigricans*, *S. aureus*, and *Vibrio cholera*	10 µg/mL	[15]
Chitosan	100–200	*E. coli*, *S. aureus*	2 µg/mL	[22]
Polymethyl methacrylate (PMMA)	-	*Acinetobactor baumannii* C78 and C80, *P. aeruginosa* RRLP1 and RRLP2	17 µg/mL	[95]
Cellulose	50–200	*E. coli* ATCC 25,922	1.15 mg/mL	[96]
Our results	30–50	*E. coli*	1 µg/mL	-

## Data Availability

The raw data supporting the conclusions of this article will be made available by the authors, without undue reservation.

## References

[1] Zaman S.B., Hussain M.A., Nye R., Mehta V., Mamun K.T., Hossain N. (2017). A review on antibiotic resistance: Alarm bells are ringing. Cureus.

[2] Gudkov S.V., Burmistrov D.E., Serov D.A., Rebezov M.B., Semenova A.A., Lisitsyn A.B. (2021). Do Iron Oxide Nanoparticles Have Significant Antibacterial Properties?. Antibiotics.

[3] Gold K., Slay B., Knackstedt M., Gaharwar A.K. (2018). Antimicrobial activity of metal and metal-oxide based nanoparticles. Adv. Ther..

[4] Gabrielyan L., Badalyan H., Gevorgyan V., Trchounian A. (2020). Comparable antibacterial effects and action mechanisms of silver and iron oxide nanoparticles on *Escherichia coli* and *Salmonella typhimurium*. Sci. Rep..

[5] Kudrinskiy A.A., Ivanov A.Y., Kulakovskaya E.V., Klimov A.I., Zherebin P.M., Khodarev D.V., Le A.T., Tam L.T., Lisichkin G.V., Krutyakov Y.A. (2014). The mode of action of silver and silver halides nanoparticles against *Saccharomyces cerevisiae* cells. J. Nanopart..

[6] Liu Y., He L., Mustapha A., Li H., Hu Z.Q., Lin M. (2009). Antibacterial activities of zinc oxide nanoparticles against *Escherichia coli* O157:H7. J. Appl. Microbiol..

[7] Sirelkhatim A., Mahmud S., Seeni A., Kaus N.H.M., Ann L.C., Bakhori S.K.M., Hasan H., Mohamad D. (2015). Review on zinc oxide nanoparticles: Antibacterial activity and toxicity mechanism. Nanomicro Lett..

[8] Saha R.K., Debanath M.K., Paul B., Medhi S., Saikia E. (2020). Antibacterial and nonlinear dynamical analysis of flower and hexagon-shaped ZnO microstructures. Sci. Rep..

[9] Xie Y., He Y., Irwin P.L., Jin T., Shi X. (2011). Antibacterial activity and mechanism of action of zinc oxide nanoparticles against *Campylobacter jejuni*. Appl. Environ. Microbiol..

[10] Sihem L., Hanine D., Faiza B. (2020). Antibacterial Activity of α-Fe_2_O_3_ and α-Fe_2_O_3_@Ag nanoparticles prepared by *Urtica* leaf extract. Nanotechnol. Russ..

[11] Shkodenko L., Kassirov I., Koshel E. (2020). Metal oxide nanoparticles against bacterial biofilms: Perspectives and limitations. Microorganisms.

[12] Gavrilenko E.A., Goncharova D.A., Lapin I.N., Nemoykina A.L., Svetlichnyi V.A., Aljulaih A.A., Mintcheva N., Kulinich S.A. (2019). Comparative study of physicochemical and antibacterial properties of ZnO nanoparticles prepared by laser ablation of Zn target in water and air. Materials.

[13] Pozdnyakov A.S., Ivanova A.A., Emel’yanov A.I., Prozorova G.F. (2020). Metal-polymer Ag nanocomposites based on hydrophilic nitrogen-and sulfur-containing copolymers: Control of nanoparticle size. Russ. Chem. Bull..

[14] Li D., Chen S., Zhang K., Gao N., Zhang M., Albasher G., Shi J., Wang C. (2021). The interaction of Ag_2_O nanoparticles with *Escherichia coli*: Inhibition–sterilization process. Sci. Rep..

[15] Kakakhel S.A., Rashid H., Jalil Q., Munir S., Barkatullah B., Khan S., Ullah R., Shahat A., Mahmood H., A-Mishari A. (2019). Polymers encapsulated aspirin loaded silver oxide nanoparticles: Synthesis, characterization and its bio-applications. Sains Malays..

[16] Tripathi S., Mehrotra G.K., Dutta P.K. (2011). Chitosan–Silver oxide nanocomposite film: Preparation and antimicrobial activity. Bull. Mater. Sci..

[17] Gul S., Rehan Z.A., Khan S.A., Akhtar K., Khan M.A., Khan M.I., Rashid M.I., Asiri A.M., Khan S.B. (2017). Antibacterial PES-CA-Ag_2_O nanocomposite supported Cu nanoparticles membrane toward ultrafiltration, BSA rejection and reduction of nitrophenol. J. Mol. Liq..

[18] Rajabi A., Ghazali M.J., Mahmoudi E., Baghdadi A.H., Mohammad A.W., Mustafah N.M., Ohnmar H., Naicker A.S. (2019). Synthesis, characterization, and antibacterial activity of Ag_2_O-loaded polyethylene terephthalate fabric via ultrasonic method. Nanomaterials.

[19] Roy A., Srivastava S.K., Shrivastava S.L., Mandal A.K. (2020). Hierarchical assembly of nanodimensional silver–silver oxide physical gels controlling nosocomial infections. ACS Omega.

[20] Yakdoumi F.Z., Hadj-Hamou A.S. (2020). Effectiveness assessment of TiO_2_-Al_2_O_3_ nano-mixture as a filler material for improvement of packaging performance of PLA nanocomposite films. J. Polym. Eng..

[21] Istirokhatun T., Yuni U., Andarani P., Susanto H. (2018). Do ZnO and Al_2_O_3_ nanoparticles improve the anti-bacterial properties of cellulose acetate-chitosan membrane?. MATEC Web Conf..

[22] Hu Z., Chan W.L., Szeto Y.S. (2008). Nanocomposite of chitosan and silver oxide and its antibacterial property. J. Appl. Polym. Sci..

[23] Hu Z., Zhang J., Chan W.L., Szeto Y.S. (2006). Suspension of silver oxide nanoparticles in chitosan solution and its antibacterial activity in cotton fabrics. MRS Online Proc. Libr..

[24] Sikora P., Augustyniak A., Cendrowski K., Nawrotek P., Mijowska E. (2018). Antimicrobial activity of Al_2_O_3_, CuO, Fe_3_O_4_, and ZnO nanoparticles in scope of their further application in cement-based building materials. Nanomaterials.

[25] Markowski A., Migdał P., Zygmunt A., Zaremba-Czogalla M., Gubernator J. (2021). Evaluation of the in vitro cytotoxic activity of ursolic acid PLGA nanoparticles against pancreatic ductal adenocarcinoma cell lines. Materials.

[26] Boltnarova B., Kubackova J., Skoda J., Stefela A., Smekalova M., Svacinova P., Pavkova I., Dittrich M., Scherman D., Zbytovska J. (2021). PLGA based nanospheres as a potent macrophage-specific drug delivery system. Nanomaterials.

[27] Gherasim O., Popescu-Pelin G., Florian P., Icriverzi M., Roseanu A., Mitran V., Cimpean A., Socol G. (2021). Bioactive ibuprofen-loaded PLGA coatings for multifunctional surface modification of medical devices. Polymers.

[28] Kaplan M.A., Sergienko K.V., Kolmakova A.A., Konushkin S.V., Baikin A.S., Kolmakov A.G., Sevostyanov M.A., Kulikov A.V., Ivanov V.E., Belosludtsev K.N. (2020). Development of a biocompatible PLGA polymers capable to release thrombolytic enzyme prourokinase. J. Biomater. Sci. Polym. Ed..

[29] Bazgir M., Zhang W., Zhang X., Elies J., Saeinasab M., Coates P., Youseffi M., Sefat F. (2021). Degradation and characterisation of electrospun polycaprolactone (PCL) and poly(lactic-co-glycolic acid) (PLGA) scaffolds for vascular tissue engineering. Materials.

[30] Kinne R.W., Gunnella F., Kunisch E., Heinemann S., Nies B., Maenz S., Horbert V., Illerhaus B., Huber R., Firkowska-Boden I. (2021). Performance of calcium phosphate cements in the augmentation of sheep vertebrae—An ex vivo study. Materials.

[31] Sevostyanov M.A., Baikin A.S., Sergienko K.V., Shatova L.A., Kirsankin A.A., Baymler I.V., Shkirin A.V., Gudkov S.V. (2020). Biodegradable stent coatings on the basis of PLGA polymers of different molecular mass, sustaining a steady release of the thrombolityc enzyme streptokinase. React. Funct. Polym..

[32] Lin L.-H., Lee H.-P., Yeh M.-L. (2020). Characterization of a Sandwich PLGA-Gallic Acid-PLGA Coating on Mg Alloy ZK60 for Bioresorbable Coronary Artery Stents. Materials.

[33] Sankar R., Kanchi S.S., Vilwanathan R. (2020). Incorporated plant extract fabricated silver/poly-D,l-lactide-co-glycolide nanocomposites for antimicrobial based wound healing. Spectrochim. Acta A Mol. Biomol. Spectrosc..

[34] Ankush P., Gurpreet K., Shikha K., Vipasha S., Shilpee S., Rajat S., Shweta S. (2019). Green chemistry mediated synthesis of PLGA-Silver nanocomposites for antibacterial synergy: Introspection of formulation parameters on structural and bactericidal aspects. React. Funct. Polym..

[35] Almajhdi F.N., Fouad H., Khalil K.A., Awad H.M., Mohamed S.H., Elsarnagawy T., Albarrag A.M., Al-Jassir F.F., Abdo H.S. (2014). In-vitro anticancer and antimicrobial activities of PLGA/silver nanofiber composites prepared by electrospinning. J. Mater. Sci. Mater. Med..

[36] Baikin A.S., Kolmakov A.G., Shatova L.A., Nasakina E.O., Sharapov M.G., Baymler I.V., Gudkov S.V., Sevostyanov M.A. (2019). Polylactide-based stent coatings: Biodegradable polymeric coatings capable of maintaining sustained release of the thrombolytic enzyme prourokinase. Materials.

[37] Hosseinpour-Mashkani S.M., Ramezani M. (2014). Silver and silver oxide nanoparticles: Synthesis and characterization by thermal decomposition. Mater. Lett..

[38] Scavone M., Armentano I., Fortunati E., Cristofaro F., Mattioli S., Torre L., Kenny J.M., Imbriani M., Arciola C.R., Visai L. (2016). Antimicrobial properties and cytocompatibility of PLGA/Ag nanocomposites. Materials.

[39] Armentano I., Fortunati E., Latterini L., Rinaldi S., Saino E., Visai L., Elisei F., Kenny J.M. (2010). Biodegradable PLGA matrix nanocomposite with silver nanoparticles: Material properties and bacteria activity. J. Nanostruct. Polym. Nanocompos..

[40] Lee W.F., Tsao K.T. (2010). Effect of silver nanoparticles content on the various properties of nanocomposite hydrogels by in situ polymerization. J. Mater. Sci..

[41] Lyutakov O., Kalachyova Y., Solovyev A., Vytykacova S., Svanda J., Siegel J., Ulbrich P., Svorcik V. (2015). One-step preparation of antimicrobial silver nanoparticles in polymer matrix. J. Nanopart. Res..

[42] Fortunati E., Mattioli S., Visai L., Imbriani M., Fierro J.L., Kenny J.M., Armentano I. (2013). Combined effects of Ag nanoparticles and oxygen plasma treatment on PLGA morphological, chemical, and antibacterial properties. Biomacromolecules.

[43] Amendola V., Meneghetti M. (2013). What controls the composition and the structure of nanomaterials generated by laser ablation in liquid solution?. Phys. Chem. Chem. Phys..

[44] Chemin A., Fawaz M.W., Amans D. (2021). Investigation of the blast pressure following laser ablation at a solid-fluid interface using shock waves dynamics in air and in water. Appl. Surf. Sci..

[45] Al-Kattan A., Grojo D., Drouet C., Mouskeftaras A., Delaporte P., Casanova A., Robin J.D., Magdinier F., Alloncle P., Constantinescu C. (2021). Short-Pulse Lasers: A Versatile Tool in Creating Novel Nano-/Micro-Structures and Compositional Analysis for Healthcare and Wellbeing Challenges. Nanomaterials.

[46] Kusoglu I.M., Huber F., Doñate-Buendía C., Rosa Ziefuss A., Gökce B., Sehrt J.T., Kwade A., Schmidt M., Barcikowski S. (2021). Nanoparticle Additivation Effects on Laser Powder Bed Fusion of Metals and Polymers—A Theoretical Concept for an Inter-Laboratory Study Design All Along the Process Chain, Including Research Data Management. Materials.

[47] Chausov D.N., Burmistrov D.E., Kurilov A.D., Bunkin N.F., Astashev M.E., Simakin A.V., Vedunova M.V., Gudkov S.V. (2021). New Organosilicon Composite Based on Borosiloxane and Zinc Oxide Nanoparticles Inhibits Bacterial Growth, but Does Not Have a Toxic Effect on the Development of Animal Eukaryotic Cells. Materials.

[48] Zhilnikova M., Barmina E., Pavlov I., Vasiliev A., Shafeev G. (2021). Laser fragmentation of Ag_2_O micropowder in water. J. Phys. Chem. Solids.

[49] Ivanyuk V.V., Shkirin A.V., Belosludtsev K.N., Dubinin M.V., Kozlov V.A., Bunkin N.F., Dorokhov A.S., Gudkov S.V. (2020). Influence of fluoropolymer film modified with nanoscale photoluminophor on growth and development of plants. Front. Phys..

[50] Sarimov R.M., Binhi V.N., Matveeva T.A., Penkov N.V., Gudkov S.V. (2021). Unfolding and aggregation of lysozyme under the combined action of dithiothreitol and guanidine hydrochloride: Optical studies. Int. J. Mol. Sci..

[51] Gudkov S.V., Simakin A.V., Sarimov R.M., Kurilov A.D., Chausov D.N. (2021). Novel Biocompatible with Animal Cells Composite Material Based on Organosilicon Polymers and Fullerenes with Light-Induced Bacteriostatic Properties. Nanomaterials.

[52] In Pyo Park P., Jonnalagadda S. (2006). Predictors of glass transition in the biodegradable poly-lactide and poly-lactide-co-glycolide polymers. J. Appl. Polym. Sci..

[53] Shtarkman I.N., Gudkov S.V., Chernikov A.V., Bruskov V.I. (2008). Effect of amino acids on X-ray-induced hydrogen peroxide and hydroxyl radical formation in water and 8-oxoguanine in DNA. Biochemistry.

[54] Chernikov A.V., Gudkov S.V., Shtarkman I.N., Bruskov V.I. (2007). Oxygen effect in heat-mediated damage to DNA. Biofizika.

[55] Gudkov S.V., Lyakhov G.A., Pustovoy V.I., Shcherbakov I.A. (2019). Influence of mechanical effects on the hydrogen peroxide concentration in aqueous solutions. Phys. Wave Phenom..

[56] Bruskov V.I., Chernikov A., Gudkov S.V., Masalimov Z.K. (2003). Heat-induced activation of reducing properties of sea-water anions. Biofizika.

[57] Baimler I.V., Simakin A.V., Uvarov O.V., Volkov M., Gudkov S.V. (2020). Generation of hydroxyl radicals during laser breakdown of aqueous solutions in the presence of Fe and Cu nanoparticles of different sizes. Phys. Wave Phenom..

[58] Gudkov S.V., Garmash S.A., Shtarkman I.N., Chernikov A.V., Karp O.E., Bruskov V.I. (2010). Long-lived protein radicals induced by X-ray irradiation are the source of reactive oxygen species in aqueous medium. Dokl. Biochem. Biophys..

[59] Gudkov S.V., Shtarkman I.N., Chernikov A.V., Usacheva A., Bruskov V.I. (2007). Guanosine and inosine (riboxin) eliminate the long-lived protein radicals induced X-ray radiation. Dokl. Biochem. Biophys..

[60] Sharapov M.G., Novoselov V.I., Penkov N.V., Fesenko E.E., Vedunova M.V., Bruskov V.I., Gudkov S.V. (2019). Protective and adaptogenic role of peroxiredoxin 2 (Prx2) in neutralization of oxidative stress induced by ionizing radiation. Free Radic. Biol. Med..

[61] Ivanov V.E., Usacheva A.M., Chernikov A.V., Bruskov V.I., Gudkov S.V. (2017). Formation of long-lived reactive species of blood serum proteins induced by low-intensity irradiation of helium-neon laser and their involvement in the generation of reactive oxygen species. J. Photochem. Photobiol. B Biol..

[62] Gudkov S.V., Guryev E.L., Gapeyev A.B., Sharapov M.G., Bunkin N.F., Shkirin A.V., Zabelina T.S., Glinushkin A.P., Sevost’yanov M.A., Belosludtsev K.N. (2019). Unmodified hydrated C_60_ fullerene molecules exhibit antioxidant properties, prevent damage to DNA and proteins induced by reactive oxygen species and protect mice against injuries caused by radiation-induced oxidative stress. Nanomedicine.

[63] Burmistrov D.E., Yanykin D.V., Paskhin M.O., Nagaev E.V., Efimov A.D., Kaziev A.V., Ageychenkov D.G., Gudkov S.V. (2021). Additive Production of a Material Based on an Acrylic Polymer with a Nanoscale Layer of Zno Nanorods Deposited Using a Direct Current Magnetron Discharge: Morphology, Photoconversion Properties, and Biosafety. Materials.

[64] Barkhudarov E.M., Kossyi I.A., Anpilov A.M., Ivashkin P.I., Artem’ev K.V., Moryakov I.V., Misakyan M.A., Christofi N., Burmistrov D.E., Smirnova V.V. (2020). New nanostructured carbon coating inhibits bacterial growth, but does not influence on animal cells. Nanomaterials.

[65] Gudkov S.V., Simakin A.V., Konushkin S.V., Ivannikov A.Y., Nasakina E.O., Shatova L.A., Kolmakov A.G., Sevostyanov M.A. (2020). Preparation, structural and microstructural characterization of Ti–30Nb–10Ta–5Zr alloy for biomedical applications. J. Mater. Res. Technol..

[66] Sevost’yanov M.A., Nasakina E.O., Baikin A.S., Sergienko K.V., Konushkin S.V., Kaplan M.A., Seregin A.V., Leonov A.V., Kozlov V.A., Shkirin A.V. (2018). Biocompatibility of new materials based on nano-structured nitinol with titanium and tantalum composite surface layers: Experimental analysis in vitro and in vivo. J. Mater. Sci. Mater. Med..

[67] Konushkin S.V., Sergiyenko K.V., Nasakina E.O., Leontyev V.G., Kuznetsova O.G., Titov D.D., Tsareva A.M., Dormidontov N.A., Kirsankin A.A., Kannykin S.V. (2020). Study of the physicochemical and biological properties of the new promising Ti–20Nb–13Ta–5Zr alloy for biomedical applications. Mater. Chem. Phys..

[68] Nahrawy A.M.E., Abou Hammad A.B., Abdel-Aziz M.S., Wassel A.R. (2019). Spectroscopic and Antimicrobial Activity of Hybrid Chitosan/Silica Membranes doped with Al_2_O_3_ Nanoparticles. Silicon (Online).

[69] Saeb A.T., Alshammari A.S., Al-Brahim H., Al-Rubeaan K.A. (2014). production of silver nanoparticles with strong and stable antimicrobial activity against highly pathogenic and multidrug resistant bacteria. Sci. World J..

[70] Ignatyev P.S., Indukaev K.V., Osipov P.A., Sergeev I.K. (2013). Laser interference microscopy for nanobiotechnologies. Biomed. Eng..

[71] Chausov D.N., Kurilov A.D., Kucherov R.N., Simakin A.V., Gudkov S.V. (2020). Electro-optical performance of nematic liquid crystals doped with gold nanoparticles. J. Phys. Condens. Matter.

[72] Khan R.A.A., Chen X., Qi H.K., Huang J.H., Luo M.B. (2021). A novel shift in the glass transition temperature of polymer nanocomposites: A molecular dynamics simulation study. Phys. Chem. Chem. Phys..

[73] Laouini S.E., Bouafia A., Soldatov A.V., Algarni H., Tedjani M.L., Ali G.A.M., Barhoum A. (2021). Green Synthesized of Ag/Ag_2_O Nanoparticles Using Aqueous Leaves Extracts of *Phoenix dactylifera* L. and Their Azo Dye Photodegradation. Membranes.

[74] Fu P.P., Xia Q., Hwang H.M., Ray P.C., Yu H. (2014). Mechanisms of nanotoxicity: Generation of reactive oxygen species. J. Food Drug Anal..

[75] Premanathan M., Karthikeyan K., Jeyasubramanian K., Manivannan G. (2011). Selective toxicity of ZnO nanoparticles toward Gram-positive bacteria and cancer cells by apoptosis through lipid peroxidation. Nanomedicine.

[76] Valko M., Rhodes C., Moncol J., Izakovic M., Mazur M. (2006). Free radicals, metals and antioxidants in oxidative stress-induced cancer. Chem. Biol. Interact..

[77] Shim M.S., Xia Y. (2013). A Reactive oxygen species (ROS)-responsive polymer for safe, efficient, and targeted gene delivery in cancer cells. Angew. Chem..

[78] Na Y., Lee J.S., Woo J., Ahn S., Lee E., Choi W.I., Sung D. (2020). Reactive oxygen species (ROS)-responsive ferro-cene-polymer-based nanoparticles for controlled release of drugs. J. Mater. Chem. B.

[79] Bruskov V.I., Karp O.E., Garmash S.A., Shtarkman I.N., Chernikov A.V., Gudkov S.V. (2012). Prolongation of oxidative stress by long-lived reactive protein species induced by X-ray radiation and their genotoxic action. Free Radic. Res..

[80] Popovich I.G., Voitenkov B.O., Anisimov V.N., Ivanov V.T., Mikhaleva I.I., Zabezhinski M.A., Alimova I.N., Baturin D.A., Zavarzina N.Y., Rosenfeld S.V. (2003). Effect of delta-sleep inducing peptide-containing preparation Deltaran on biomarkers of aging, life span and spontaneous tumor incidence in female SHR mice. Mech. Ageing Dev..

[81] Yin J.J., Fu P.P., Lutterodt H., Zhou Y.T., Antholine W.E., Wamer W. (2012). Dual role of selected antioxidants found in dietary supplements: Crossover between anti-and pro-oxidant activities in the presence of copper. J. Agric. Food Chem..

[82] Dwyer B.E., Raina A.K., Perry G., Smith M.A. (2004). Homocysteine and Alzheimer’s disease: A modifiable risk?. Free Radic. Biol. Med..

[83] Guerrero-Cázares H., Tzeng S.Y., Young N.P., Abutaleb A.O., Quiñones-Hinojosa A., Green J.J. (2014). Green biodegradable polymeric nanoparticles show high efficacy and specificity at DNA delivery to human glioblastoma in vitro and in vivo. ACS Nano.

[84] Bruskov V.I., Malakhova L.V., Masalimov Z.K., Chernikov A.V. (2002). Heat-induced formation of reactive oxygen species and 8-oxoguanine, a biomarker of damage to DNA. Nucleic Acids Res..

[85] Kneuer C., Sameti M., Bakowsky U., Schiestel T., Schirra H., Schmidt H., Lehr C.M. (2000). A Nonviral DNA delivery system based on surface modified silica-nanoparticles can efficiently transfect cells in vitro. Bioconjug. Chem..

[86] Zhang T., Lin K., Jiang H., Gao Y., Ming C., Ruan B., Ma J., Li C., Lou F., Yang Y. (2017). Core-shell lipid polymer nanoparticles for combined chemo and gene therapy of childhood head and neck cancers. Oncol. Rep..

[87] Cohen H., Levy R.J., Gao J., Fishbein I., Kousaev V., Sosnowski S., Slomkowski S., Golomb G. (2000). Sustained delivery and expression of DNA encapsulated in polymeric nanoparticles. Gene Ther..

[88] Nehra P., Chauhan R.P., Garg N., Verma K. (2017). Antibacterial and antifungal activity of chitosan coated iron oxide nanoparticles. Br. J. Biomed. Sci..

[89] Karp O.E., Gudkov S.V., Garmash S.A., Shtarkman I.N., Chernikov A.V., Bruskov V.I. (2010). Genotoxic effect of long-lived reactive protein radicals in vivo generated by X-ray irradiation. Dokl. Biochem. Biophys..

[90] Bruskov V.I., Popova N.R., Ivanov V.E., Karp O.E., Chernikov A.V., Gudkov S.V. (2014). Formation of long-lived reactive species of blood serum proteins by the action of heat. Biochem. Biophys. Res. Commun..

[91] Thukkaram M., Sitaram S., Kannaiyan S.K., Subbiahdoss G. (2014). Antibacterial efficacy of iron-oxide nanoparticles against biofilms on different biomaterial surfaces. Int. J. Biomater..

[92] Davis N., Curry A., Gambhir A.K., Panigrahi H., Walker C.R.C., Wilkins E.G.L., Worsley M.A., Kay P.R. (1999). Intraoperative bacterial contamination in operations for joint replacement. J. Bone Jt. Surg..

[93] Hughes S.P.F., Anderson F.M. (1999). Infection in the operating room. J. Bone Jt. Surg..

[94] Titov V., Nikitin D., Naumova I., Losev N., Lipatova I., Kosterin D., Pleskunov P., Perekrestov R., Sirotkin N., Khlyustova A. (2020). Dual-mode solution plasma processing for the production of chitosan/Ag composites with the antibacterial effect. Materials.

[95] Aazem I., Rathinam P., Pillai S., Honey G., Vengellur A., Bhat S.G., Sailaja G.S. (2021). Active bayerite underpinned Ag_2_O/Ag: An efficient antibacterial nanohybrid combating microbial contamination. Metallomics.

[96] Sboui M., Lachheb H., Bouattour S., Gruttadauria M., La Parola V., Liotta L.F., Boufi S. (2021). TiO_2_/Ag_2_O immobilized on cellulose paper: A new floating system for enhanced photocatalytic and antibacterial activities. Environ. Res..

